# Supplementary material for: Comparative Evaluation of the Performance of Sterile Filters for Bioburden Protection and Final Fill in Biopharmaceutical Processes
Source: Membranes (Basel). 2022 May 16;12(5):524. doi: 10.3390/membranes12050524 (PMC9143324; doi:10.3390/membranes12050524)
Supplement: Supplementary file 1 [file membranes-12-00524-s001.zip › membranes-1699123-supplementary.pdf]

membranes

MDPI

Supplementary Information

Comparative Evaluation of the Performance of Sterile Filters for Bioburden Protection and Final Fill in Biopharmaceutical Processes

Jimin Na [1], Dongwoo Suh [2], Young Hoon Cho [3,4,*] and Youngbin Baek [1,*]

[1] Department of Biological Engineering, Inha University, 100 Inha-ro, Michuhol-gu, Incheon 22212, Korea; jmna0227@gmail.com
[2] School of Chemical and Biological Engineering, College of Engineering, Institute of Chemical Process (ICP), Seoul National University (SNU), 1 Gwanak-ro, Gwanak-gu, Seoul 08826, Korea; dwsuh1@snu.ac.kr
[3] Green Carbon Research Center, Korea Research Institute of Chemical Technology (KRICT), 141 Gajeong-ro, Yuseong-gu, Daejeon 34114, Korea
[4] Department of Advanced Materials and Chemical Engineering, University of Science & Technology (UST), Yuseong-gu, Daejeon 34113, Korea
* Correspondence: yhcho@krict.re.kr (Y.H.C.); ybbaek@inha.ac.kr (Y.B.); Tel.: +82-42-860-7684 (Y.H.C.); +82-32-860-7516 (Y.B.)

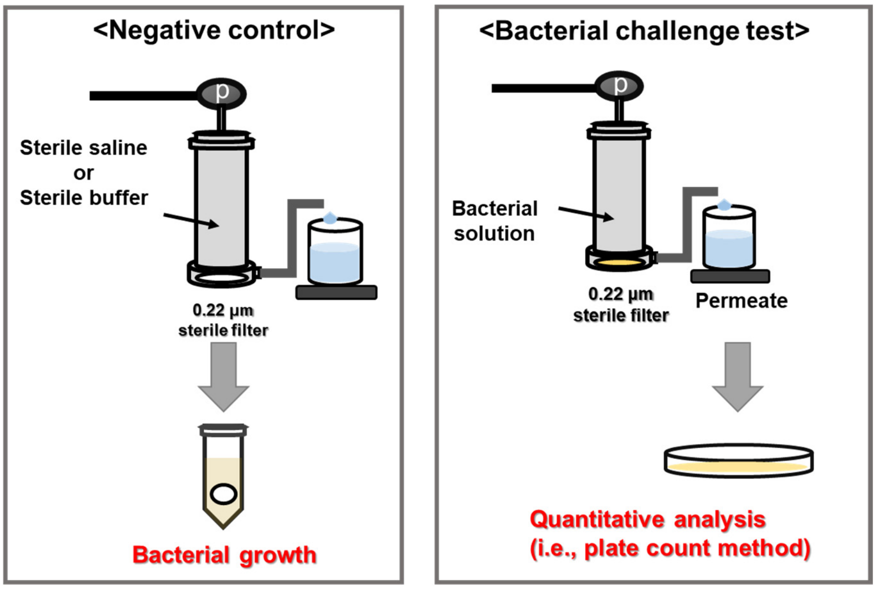

Figure S1. Experimental procedure of bacterial challenge test with negative control.

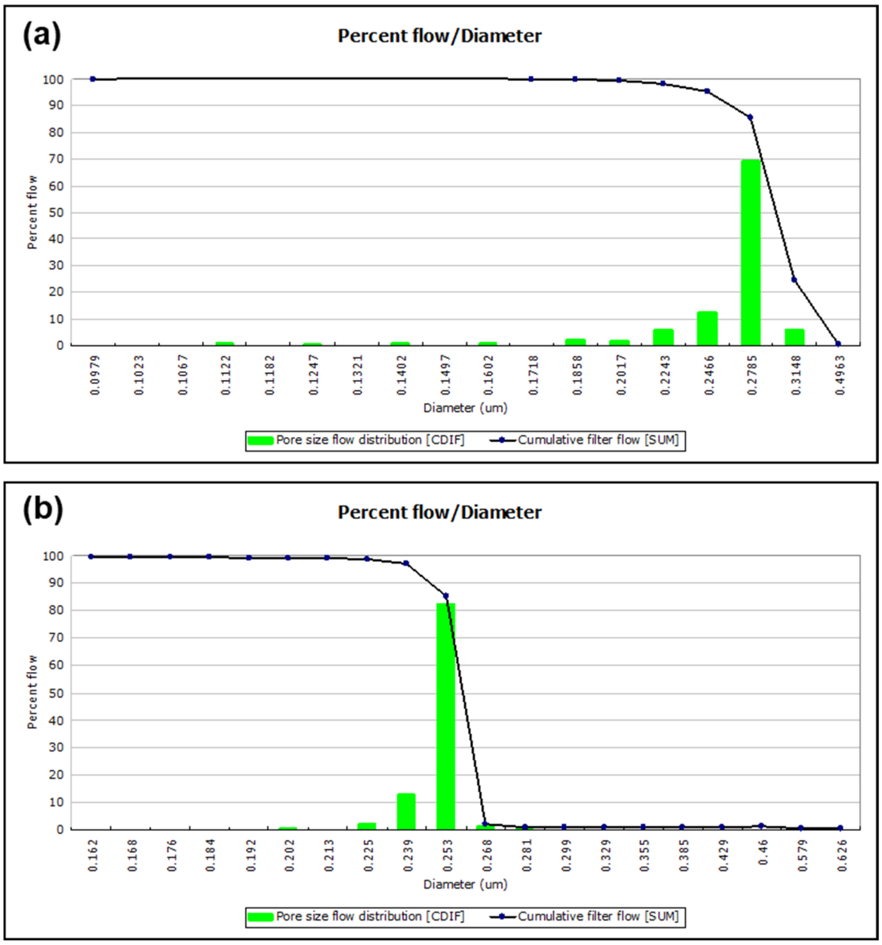

Figure S2. Gas–liquid porometry for (a) filter A and (b) filter B.

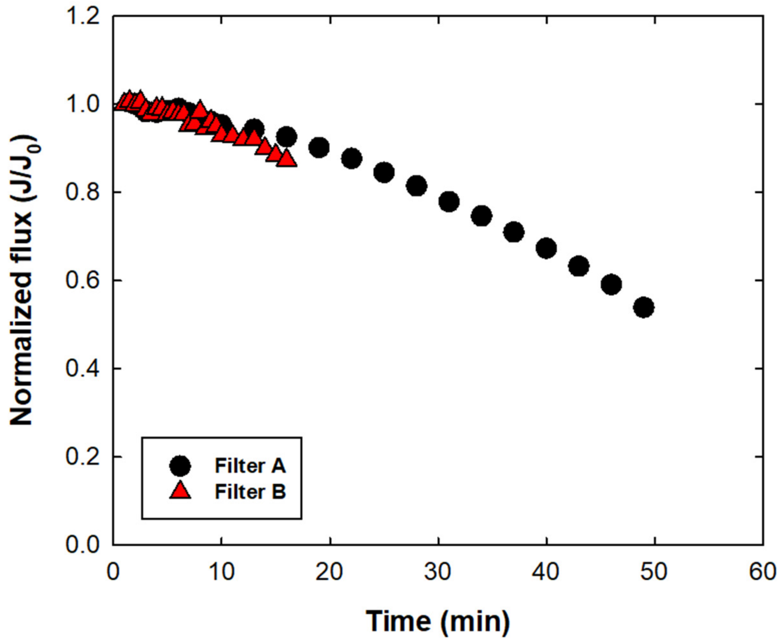

Figure S3. Normalized flux versus time plot for LB broth filtration using filters A and B.

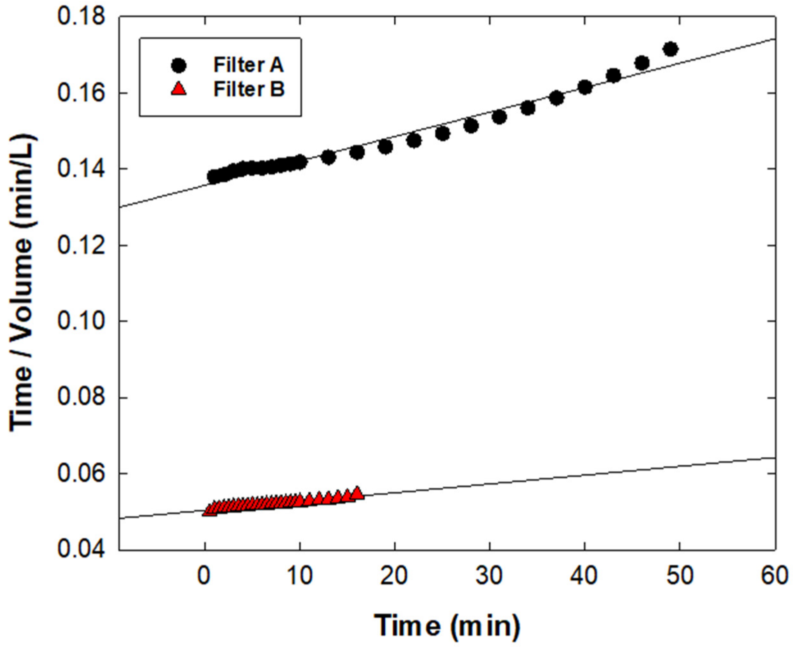

Figure S4. Time versus time/volume plot for calculating maximum permeate volume (V_{max}) of filters A and B.

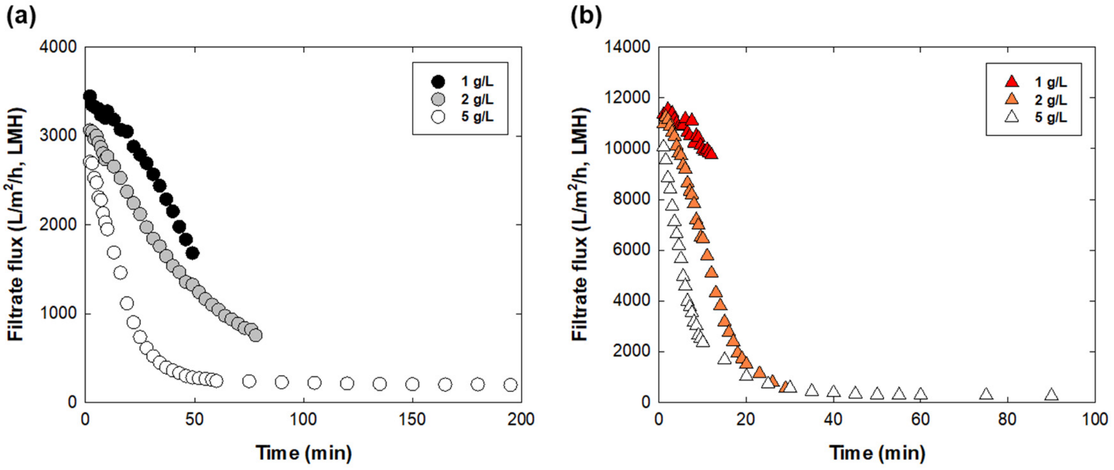

Figure S5. Filtrate flux as function of operation time at different BSA concentrations (1, 2, and 5 g/L) using (a) filter A and (b) filter B for final fill.

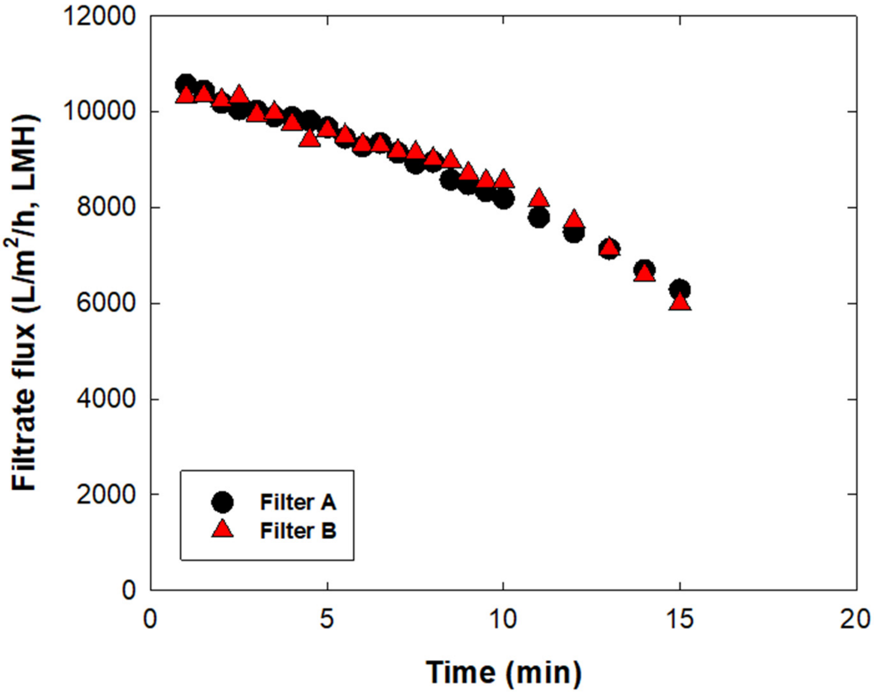

Figure S6. Filtrate flux as function of operation time for 2 g/L of BSA solution filtration at same initial flux (approximately 10,500 LMH) with adjusted operating pressure at 1.5 bar for filter A.
